# Sir Henry Burdett's Inspection Tour

**Published:** 1909-10-16

**Authors:** 


### SIR HENRY BURDETT'S INSPECTION TOUR.

The following notice appears in last week's *Truth* :—"The Hospital commences in its current issue the publication of a series of reports on the hospitals of the United Kingdom—the result of a personal inspection which Sir Henry Burdett is carrying out. These reports are likely to be of considerable public interest and utility. Nothing of the sort has been attempted since 1863, when an exhaustive inspection was undertaken, at the instance of the Privy Council, by Dr. Bristowe and Mr. Timothy Holmes, whose reports were printed by the Government. There is room for systematic inspection of this kind in the interests alike of patients, subscribers, and hospital authorities, lay and medical. My own impression is that it should be undertaken officially, and possibly it will be some day. In the meantime Sir Henry Burdett renders a real service to the public by undertaking the work voluntarily—a task for which he is probably better fitted than anyone living by practical knowledge and long experience of hospital administration."

